# A Multicenter Evaluation of Ceftolozane/Tazobactam Treatment Outcomes in Immunocompromised Patients With Multidrug-Resistant *Pseudomonas aeruginosa* Infections

**DOI:** 10.1093/ofid/ofab089

**Published:** 2021-03-06

**Authors:** Delaney E Hart, Jason C Gallagher, Laura A Puzniak, Elizabeth B Hirsch, C/T Alliance to deliver Real-world Evidence (CARE), Aiman Bandali, Kirthana R Beaulac, Tiffany E Bias, Kenneth Biason, Christopher M Bland, Kimberly Boeser, Saira Chaudhry, Kimberly C Claeys, Ashley L Cubillos, Brandon Dionne, Deepali Dixit, Claudine El-Beyrouty, Abdulrahman Elabor, Elizabeth Gancher, Yi Guo, Nicole Harrington, Emily L Heil, Jon Hiles, Bruce M Jones, Madeline A King, Xiaoning Lu, Monica V Mahoney, Dorothy McCoy, Erin K McCreary, Esther Molnar, Ashley Piche, Janet K Raddatz, Lynette Richards, Nidhi Saraiya, Michael J Satlin, Jin Suh, Abinash Virk, Nikunj M Vyas, Daohai Yu

**Affiliations:** 1 University of Minnesota College of Pharmacy, Minneapolis, Minnesota, USA; 2 Temple University School of Pharmacy, Philadelphia, Pennsylvania, USA; 3 Merck & Co., Inc., Kenilworth, New Jersey, USA

**Keywords:** ceftolozane/tazobactam, immunocompromised, multidrug-resistant, *P. aeruginosa*, pneumonia

## Abstract

**Background:**

Real-world data assessing outcomes of immunocompromised patients treated with ceftolozane/tazobactam (C/T) are limited. This study evaluated treatment and clinical outcomes of immunocompromised patients receiving C/T for multidrug-resistant (MDR) *Pseudomonas aeruginosa*.

**Methods:**

This was a 14-center retrospective cohort study of adult immunocompromised inpatients treated for ≥24 hours with C/T for MDR *P. aeruginosa* infections. Patients were defined as immunocompromised if they had a history of previous solid organ transplant (SOT), disease that increased susceptibility to infection, or received immunosuppressive therapies. The primary outcomes were all-cause 30-day mortality and clinical cure.

**Results:**

Sixty-nine patients were included; 84% received immunosuppressive agents, 68% had a history of SOT, and 29% had diseases increasing susceptibility to infection. The mean patient age was 57 ± 14 years, and the median (interquartile range) patient Acute Physiology and Chronic Health Evaluation II and Charlson Comorbidity Index scores were 18 (13) and 5 (4), respectively, with 46% receiving intensive care unit care at C/T initiation. The most frequent infection sources were respiratory (56%) and wound (11%). All-cause 30-day mortality was 19% (n = 13), with clinical cure achieved in 47 (68%) patients. Clinical cure was numerically higher (75% vs 30%) in pneumonia patients who received 3-g pneumonia regimens vs 1.5-g regimens.

**Conclusions:**

Of 69 immunocompromised patients treated with C/T for MDR *P. aeruginosa*, clinical cure was achieved in 68% and mortality was 19%, consistent with other reports on a cross-section of patient populations. C/T represents a promising agent for treatment of *P. aeruginosa* resistant to traditional antipseudomonal agents in this high-risk population.

Ceftolozane/tazobactam (C/T) was approved for use in the United States in 2014 [1]. C/T is approved for treatment of complicated urinary tract infections (cUTIs) including pyelonephritis using a 1.5-g-based regimen and for complicated intra-abdominal infections in combination with metronidazole. In 2019, C/T was also approved for hospital-acquired and ventilator-associated bacterial pneumonia (HABP/VABP) at an increased 3-g-based regimen [1–3]. C/T has demonstrated activity against multidrug-resistant (MDR) *Pseudomonas aeruginosa* and ESBL-producing Enterobacterales via numerous in vitro studies [4–7].

Complex patient populations are often excluded from phase 3 clinical trials to ensure homogeneity of the patient population. A large subset of patients that are often at higher risk of MDR infections includes immunocompromised patients; however, the outcomes of this patient population with novel agents are often not studied or reported in registration trials. In particular, patients with hematologic malignancies and transplant recipients have a particularly high risk of gram-negative bacteremia due to gastrointestinal mucositis, neutropenia for prolonged periods, and frequent health care exposure [8, 9]. Data analyzing the use of C/T among immunocompromised patient populations are still very limited, despite this agent being in clinical use since 2014. Notably, most of the publications include small sample sizes, case reports, and case reviews [10–15]. A recent review of 7 adult patients with hematologic malignancies or hematopoietic cell transplant recipients treated with C/T demonstrated a 100% 30-day survival and 71.4% clinical cure rate [10]. Several of the larger recent cohort studies evaluating outcomes of patients treated with C/T for MDR *P. aeruginosa* infections included limited patients (21% or less) with immunocompromising conditions.

In light of the limited data available for this patient population, we aimed to evaluate treatment patterns and clinical outcomes of immunocompromised patients treated with C/T for multidrug-resistant *P. aeruginosa* infections in an effort to better understand the place in therapy for this agent among this high-risk patient group.

## METHODS

### Study Design and Data Collection

This was a multicenter, retrospective cohort study of adult (≥18 years) immunocompromised in patients treated for MDR *P. aeruginosa* from any infection source between March 2015 and July 2018. Data were collected from 14 centers across the United States. Inclusion criteria included treatment with C/T for ≥24 hours, a positive index culture for MDR *P. aeruginosa*, and immunocompromised status at the time of treatment. Patients were considered immunocompromised if they met any of the following criteria: previous solid organ transplant (SOT) recipient; having a disease that increased susceptibility to infection (leukemia, lymphoma, diffuse metastatic cancer); or receipt of therapy that increased susceptibility to infection including immunosuppressive agents, chemotherapy, radiation, steroids at doses capable of immunosuppression (≥10 mg of prednisone for ≥1 month before hospitalization or >15 mg/kg/d of hydrocortisone or >3 mg/kg/d of methylprednisolone for >5 days). Lack of C/T susceptibility data for *P. aeruginosa* index culture(s) was not an exclusion criterion.

Clinical and microbiologic data for this study were entered into a standardized data collection form using REDCap, a secure, web-based application designed to support data capture for research studies, providing (1) an intuitive interface for validated data entry; (2) audit trails for tracking data manipulation and export procedures; (3) automated export procedures for seamless data downloads to common statistical packages; and (4) procedures for importing data from external sources [16, 17]. Data collected included baseline demographics, infection type and source, antimicrobial use and duration, and clinical outcomes. The clinical decisions regarding infection treatment and antimicrobial selection were at the discretion of the attending physicians at each respective hospital. Dosing of C/T was selected by the ordering provider at each individual site. Pneumonia-dosed C/T was defined as the Food and Drug Administration (FDA)–approved dosing for HABP/VABP at 3 g intravenously every 8 hours or renally adjusted per package insert (further referred to as “pneumonia dosing/dosed” within the manuscript). Severity of illness was assessed by capturing Acute Physiology and Chronic Health Evaluation (APACHE) II scores, and degree of comorbid illness was assessed using the Charlson comorbidity index (CCI). These scores were calculated based on patient laboratory values on day 1 of suspected infection.

### Patient Consent Statement

The study was approved at each center by the designated institutional review board. Due to the retrospective study design, the requirement for signed patient consent was waived.

### Definitions

Index cultures were defined as the first culture positive for MDR *P. aeruginosa* for which C/T therapy was prescribed; *P. aeruginosa* isolates were characterized as MDR if nonsusceptible to ≥3 classes of antipseudomonal agents [18]. The index events were defined as infection or suspected infection/event for which C/T therapy was prescribed. In patients with polymicrobial infections, the first negative culture was defined as clearance of the MDR *P. aeruginosa* for which C/T was initiated. Infection was defined as per the US Centers for Disease Control and Prevention criteria for each source as assessed by individual investigators.

### Statistical Analysis

Baseline patient characteristics and treatment parameters were compared between treatment groups using the Student *t* test and Kruskal-Wallis test for continuous variables and the Fisher exact test for categorical variables. Classification and regression tree (CART) analysis was used to identify the 30-day mortality split in APACHE II scores to assess which patients may be at a greater risk for mortality. Statistical significance was set at a level of *P* < .05. Statistical analyses were performed using Systat, version 13.0 (Systat Software, Inc., San Jose, CA, USA).

### Outcomes

The primary outcomes were all-cause 30-day mortality and clinical cure. Clinical cure was assessed in patients who received continuous C/T therapy for ≥72 hours and was defined as no escalation of/additional antipseudomonal antibiotic therapy and improved signs and symptoms from baseline to end of therapy, including defervescence and discharge notations indicating stability of infection. In patients with pneumonia, primary outcomes were compared between those who received approved pneumonia (3-g-based regimen) and nonpneumonia dosing. Secondary outcomes included length of C/T therapy and total length of hospital stay. Outcomes were assessed by site investigators and confirmed by at least 1 other investigator (E.B.H., D.E.H., or J.C.G.).

## RESULTS

### Clinical Characteristics

A total of 69 patients were included; 58 (84%) had received immunosuppressive agents, 47 (66%) had a history of SOT, and 20 (29%) had diseases that increased susceptibility to infection including leukemia (9%), lymphoma (4%), and diffuse metastatic cancer (13%) (Table 1). The mean patient age was 57 ± 14 years, and common comorbidities included chronic pulmonary disease (46%), chronic kidney disease (41%), and diabetes (25%). The median (interquartile range [IQR]) patient APACHE II and Charlson Comorbidity Index scores were 18 (13) and 5 (4), respectively, with 32 (46%) receiving intensive care unit (ICU) care at C/T initiation. The most frequent infection sources were respiratory (57%) and wound (12%). Four patients had multiple infection sources: 1 had a CNS, bone/joint, and wound infection; 1 had pneumonia and wound infection; 2 had concurrent CNS and bone/joint infections.

**Table 1.** Baseline Characteristics of the Patients

Characteristic	Total (n = 69)
Age, mean ± SD, y	57 ± 14
In ICU on day 1, No. (%)	32 (46)
APACHE II score, median (IQR)	18 (13)
Charlson comorbidity index, median (IQR)	5 (4)
Immunocompromised type,^a^ No. (%)	
Receiving immunosuppressive agents	58 (84)
Solid organ transplant recipient	47 (68)
Immunocompromising disease state^b^	20 (29)
Leukemia	6 (9)
Lymphoma	3 (4)
Diffuse metastatic cancer	9 (13)
Comorbidities, No. (%)	
Chronic pulmonary disease	32 (46)
Chronic kidney disease	28 (41)
Diabetes	17 (25)
Myocardial infarction	10 (14)
Heart failure	10 (14)
Peptic ulcer disease	9 (13)
Liver dysfunction	9 (13)
Peripheral vascular disease	8 (12)
Cerebrovascular disease	5 (7)
Metastatic solid tumor	5 (7)
Cystic fibrosis	4 (6)
Hemiplegia/paraplegia	2 (3)
Infection source,^c^ No. (%)	
Pneumonia	39 (57)
Wound	8 (12)
Intra-abdominal	6 (10)
Primary bloodstream infection	6 (10)
Urinary tract	6 (10)
Bone/joint	4 (6)
Central nervous system	3 (4)
Concurrent antibiotics, No. (%)	31 (45)
Aminoglycoside	15 (48)
Fluoroquinolone	9 (29)
Polymyxin	7 (23)
Beta-lactam	2 (6)

Abbreviations: APACHE, Acute Physiology and Chronic Health Evaluation; ICU, intensive care unit; IQR, interquartile range.

^a^Patients could have multiple reasons for immunocompromised classification.

^b^Two patients with unspecified disease characterized as sufficiently advanced to suppress resistance to infection, for example, leukemia, lymphoma, diffuse metastatic cancer.

^c^Patients could have multiple sources of infection.

### Treatment Characteristics

Overall, 36% of patients had a polymicrobial culture, with 45% of patients receiving combination antimicrobial therapy. The most commonly used concurrent antibiotics were aminoglycosides in 15 patients (48% of concurrent antibiotics), followed by fluoroquinolones in 9 patients (29%), polymyxins in 7 patients (23%), and beta-lactams in 2 patients (6%). Of the 39 patients with pneumonia, 28 (71.8%) received 3-g pneumonia dosing, 10 (25.6%) received 1.5-gram (nonpneumonia) dosing, and 1 patient had incomplete dosing data.

### Outcomes

**Figure 1.**
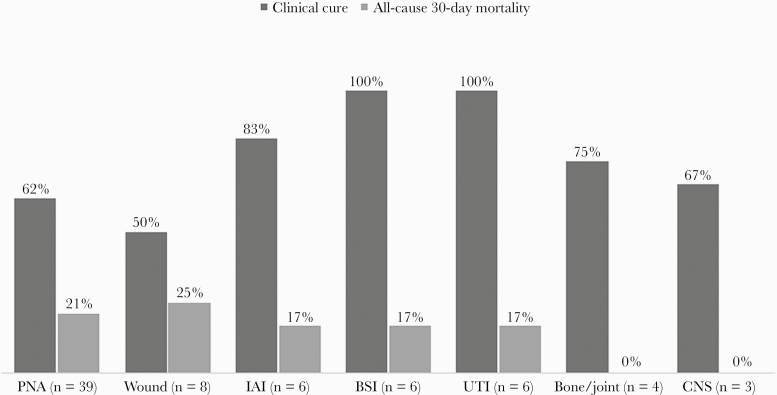
Clinical outcomes by source of infection. Abbreviations: BSI, primary bloodstream infection; CNS, central nervous system; IAI, intra-abdominal infection; PNA, pneumonia; UTI, urinary tract infection.

All-cause 30-day mortality among all patients was 19% (13/69), with clinical cure achieved in 68% (47/69) of patients (Table 2). Clinical cure and all-cause 30-day mortality rates varied by infection source, with the highest rates of clinical cure in patients with UTI (100%; 6/6) and bloodstream infections (100%; 6/6) and the lowest all-cause 30-day mortality rates in patients with central nervous system and bone/joint infections (both 0%) (Figure 1). In patients with pneumonia, clinical cure was 75% (21/28) in the 3-g pneumonia dosing group vs 30% (3/10) in the nonpneumonia dosing group, and 30-day mortality was 18% (5/28) in those who received the pneumonia-dose C/T vs 30% (3/10) in those who did not. The mean length of C/T therapy was 13 ± 10.8 days, and the median (IQR) length of hospital stay was 38 (55) days. CART analysis identified the 30-day mortality split at APACHE II score >25 (76% vs 24%; *P* = 0.002).

**Table 2.** Clinical Outcomes

Outcome	
Clinical cure, all infection sources (n = 69), No. (%)	47 (68)
Pneumonia, receiving pneumonia dosing (n = 28)	21 (75)
Pneumonia, receiving nonpneumonia dosing (n = 10)	3 (30)
30-d all-cause mortality, all infection sources (n = 69), No. (%)	13 (19)
Pneumonia, receiving pneumonia dosing (n = 28)	5 (18)
Pneumonia, receiving nonpneumonia dosing (n = 10)	3 (30)
Length of C/T therapy, mean ± SD, d	13 ± 11
Length of hospital stay, median (IQR), d	38 (54)

Abbreviations: C/T, ceftolozane/tazobactam; IQR, interquartile range.

## DISCUSSION

This 14-center study aimed to evaluate real-world treatment patterns and clinical outcomes of immunocompromised patients treated with C/T for multidrug-resistant *P. aeruginosa* infections. As a majority of current clinical data exclude immunocompromised patients or these patients make up a small subset of the studied patient population, it is pertinent to describe outcomes in this high-risk group. Patients in our cohort were characterized as immunocompromised for a variety of conditions. A majority of patients were taking immunosuppressive agents (84%), a subset had a history of SOT (68%), and a smaller subset of patients had diseases conferring susceptibility to infection such as active malignancies (29%). In addition to an immunocompromised status of all included patients, many were considered critically ill, demonstrated by a median APACHE II score of 18, with 46% of patients receiving ICU-level care upon C/T initiation. The CART analysis identifying the 30-day mortality split at APACHE II score >25 demonstrates that the most critically ill patients with a high APACHE II score were at greatest risk for mortality. Furthermore, these patients had prolonged hospital stays, as demonstrated by a median hospital length of stay of 38 days, although many factors can confound hospital length of stay in immunocompromised patients.

In the present study of 69 immunocompromised patients, morbidity and clinical cure rates were similar to previous, larger studies conducted within nonimmunocompromised patient populations. Patients receiving 1.5-g C/T dosing plus metronidazole in the ASPECT-cIAI trial had a clinical cure rate of 76.9% in patients receiving C/T, and patients treated with the 3-g C/T dose in the phase 3 ASPECT-NP clinical trial had a 28-day all-cause mortality rate of 24.0% and clinical cure rate of 54% [3, 19]. In addition to the phase 3 trials evaluating C/T, 1 of the largest studies evaluating use of C/T specifically for MDR *P. aeruginosa* reported clinical success in 73.7% of patients and 30-day mortality in 19% of patients [20]. This study included 205 patients, with a median age (IQR) of 60 (48–70) years and the most frequent infection source being pneumonia (59%). The median CCI (IQR) was 4 (3–6), and the median APACHE II score (IQR) was 19 (11–24), which was similar to the comorbidity and severity of illness of patients in the present study. Of the 205 patients, 35 (17.1%) had a history of organ transplantation and 33 (16.1%) had a history of cancer, although outcomes were not reported specific to disease states. A recent observational cohort study of C/T use for MDR or XDR *P. aeruginosa* in comparison with aminoglycoside or polymyxin included 100 patients treated with C/T. Clinical cure was observed in 81% of C/T-treated patients; of these, only 14 patients were noted to be immunosuppressed [13]. A third observational study of C/T use for treatment of MDR and XDR *P. aeruginosa* evaluated 58 patients, noting a 63.8% clinical cure rate and 27.6% 30-day mortality; however, only 7 (12%) of the included patients were reported to be immunosuppressed [13, 21]. In comparison to these larger studies, immunocompromised individuals in the present study had very similar clinical success (68%) and all-cause 30-day mortality (19%) rates.

When evaluating clinical outcomes by infection source in this cohort, clinical cure was achieved most often in patients with UTI, bloodstream infections, and intra-abdominal infections. Thirty-day all-cause mortality rates ranged from 0% to 25% overall and were lowest in patients with bone/joint infections and CNS infections; however, these groups were very small, making the data difficult to extrapolate. A primary source of pneumonia encompassed slightly over half (n = 39; 56%) of the patient cohort. Clinical cure was achieved in only 62% of these patients; however, upon analysis of clinical cure stratified by FDA-approved 3-g pneumonia dosing of C/T, clinical cure was numerically higher in those who received the appropriate pneumonia dose (75% vs 30%), and 30-day mortality was numerically lower (18% vs 30%) in the pneumonia patients receiving pneumonia dosing. This higher 3-g dose/indication was approved in 2019 while data from this retrospective cohort date back to 2015, so it is reasonable that the higher 3-g pneumonia dosing was not universally used off-indication. While this cohort is small, these results demonstrate the importance of utilizing the FDA-approved dosing of 3 g for patients with pneumonia.

Other smaller studies examining the outcomes of C/T use exclusively among immunocompromised patients have consisted mainly of case reports or small case series [10, 12]. One retrospective review of 21 patients treated with C/T for MDR *P. aeruginosa* included a large subset of immunocompromised patients, with 9 (43%) characterized as transplant recipients [14]. This study reported a 30-day all-cause mortality rate of 10% and a clinical success rate of 71%. A recent review of 6 adult patients with hematologic malignancies or hematopoietic cell transplant recipients treated with C/T monotherapy for MDR *P. aeruginosa* demonstrated a 100% 30-day survival and 71.4% clinical cure rate. Sources of infection in these patients included pneumonia (n = 3), undefined primary source (n = 3), and soft tissue infection (n = 1) [10].

To our knowledge, this is the largest cohort study examining outcomes in an immunocompromised patient population; however, it does have several limitations. Our study is limited by the relatively small sample size as well as the retrospective nature of data collection. Documented history of immunocompromising conditions, such as SOT (n = 47), was used to categorize patients having increased susceptibility to infection. However, it was not known in all cases how recent a patient’s SOT or cancer diagnosis was or what level of immunosuppressive therapy they were being treated with at the time of the index culture. Specifications regarding source control of various infections and reasons for patients not meeting the study definition of clinical cure were not collected and were therefore not assessed. Additionally, there was no control group with which to compare C/T outcomes with alternative antibiotics. However, the study was robust in the aspect that patient data were collected across 14 medical centers in various geographical areas of the United States and therefore represents a real-world approach to treatment and outcomes in this complicated patient population. Though a large proportion of patients received concurrent antimicrobials, an assessment of whether they possessed in vitro activity against the *P. aeruginosa* index isolate was not conducted. Further investigation could be warranted to assess the role of potential combination therapy and outcomes related to MDR *P. aeruginosa* infections. Among the 31 patients receiving C/T plus concurrent antibiotics, the largest proportion were treated with aminoglycosides, which are known to cause nephrotoxicity [13]. In light of the potent in vitro activity of C/T against MDR *P. aeruginosa*, use of C/T as a means to avoid use of more toxic broad-spectrum antibiotics could contribute to improved stewardship goals such as decreased antibiotic resistance and/or decreased potential for adverse effects in light of the improved safety profile of beta-lactams [7].

## CONCLUSIONS

In this cohort of 69 immunocompromised patients from 14 US centers treated with C/T, we found clinical cure (68%) and mortality (19%) rates similar to those reported in randomized clinical trials and other real-world studies. Our data help to support the conclusion that C/T appears to be a safe and effective therapy for treatment of MDR *P. aeruginosa* infections in immunocompromised patients. Clinical cure and mortality differences seen in patients with pneumonia stratified by dosing scheme underscore the need to ensure that the approved 3-g dose is being used in patients with pneumonia. Larger controlled studies are warranted to further validate these outcomes.
